# Educational insights of artificial intelligence assisted chest radiograph interpretation in lung cancer detection—pictorial review

**DOI:** 10.1093/bjrai/ubaf002

**Published:** 2025-02-20

**Authors:** Jenna R Tugwell-Allsup, Andrew England, Bethan W Owen

**Affiliations:** Radiology Department, Betsi Cadwaladr University Health Board, Bangor, Gwynedd LL57 2PW, United Kingdom; Discipline of Medical Imaging & Radiation Therapy, School of Medicine, Brookfield Health Sciences Complex, University College Cork, Cork T12 AK54, Ireland; Radiology Department, Betsi Cadwaladr University Health Board, Bangor, Gwynedd LL57 2PW, United Kingdom

**Keywords:** AI, chest X-ray, CXR, image quality, false-positive, education, artificial intelligence

## Abstract

Chest radiographs (CXRs) are one of the most complicated, yet most frequently performed imaging examinations worldwide, and are susceptible to reporting discrepancies, especially in the detection of lung cancer (LC). The use of artificial intelligence (AI) as a decision-supporting tool for CXRs has been shown to aid earlier LC detection and reduce reporting discrepancies. Nevertheless, the use of AI within radiology is still in its infancy in terms of clinical implementation. There is a need for more pragmatic evidence to enhance key stakeholders’ perceptions and understating of AI within clinical practice. Most AI studies focus on comparing sensitivity, specificity, and accuracy against a re-review of pre-selected images by radiologists, often paired with complex technical data and statistical analyses. Improved understanding and awareness of AI capabilities whilst learning from AI discrepancies could help optimize human–AI interactions and acceptance within clinical practice whilst informing future research. The aim of this educational review is to provide a visual portrayal of real-world examples of missed LC cases from within a retrospective AI study, explore the impact of image quality on AI performance, and highlight cases of AI discrepancies.

## Introduction

Chest radiographs (CXRs) are the most frequently performed imaging examinations worldwide, providing an early opportunity to detect both symptomatic and asymptomatic lung cancer (LC).^1^ CXRs can be difficult to interpret due to the superimposition of various structures of differing radiographic densities and are therefore susceptible to discrepancies in LC diagnosis.^2^ Developments in artificial intelligence (AI) have soared within radiology; evidence suggests that AI can aid observer-reporting performance whilst reducing discrepancies,^1^^,^^3^ especially in detecting lung nodules.^3–5^ Nevertheless, evidence provided within large-scale AI radiology studies often lacks accessible information that visually demonstrates AI use on patient images, thus enabling better appreciation of how AI can enhance practice. The Royal College of Radiologists^6^ further highlights in their recent guidance on AI deployment fundamentals the need for greater showcasing of real-world scenarios to enhance learning. Otherwise, the lack of awareness of how AI works in terms of its strengths and weaknesses may have adverse effects, such as a phenomenon known as “automation bias.”^7^ Bernstein et al.^7^ reported that when AI provided incorrect results, false-positive rates among the radiologists increased, highlighting the importance of recognizing common pitfalls associated with AI systems.

Providing radiologists and reporting radiographers with visual real-world examples of AI within different contexts, populations, and settings can improve appreciation of how AI can enhance their practice, empowering them to leverage AI’s benefits while being mindful of its limitations, ultimately improving patient care and advancing the field of radiology. Visually demonstrating potential discrepancies in detecting LC on CXRs can also help navigate future research requirements. These include the importance of observer decision-making and how AI affects this process in difficult cases, for example recognizing false positives (FPs) vs true positives.

Evidence continues to demonstrate the educational gap in terms of adequacy of AI knowledge within clinical practice, which varies across different generations and globally. A visual educational review of an AI software, as opposed to in-depth knowledge regarding the AI’s technical performance, would positively contributes to the current evidence.^8^^,^^9^

This pictorial review presents educational insights on interesting cases from within a recent retrospective AI LC study. These patient cases are of CXRs acquired up to 6 years prior to *known* LC diagnosis, in addition to negative controls that were evaluated retrospectively using commercially available AI software (Table 1). The AI software was trained on >12 408 abnormal CXRs with lung nodules or masses with the dataset annotated by at least 1 of 15 board-certified radiologists. Within this new work, findings from the AI software were correlated against the original clinical reports from consultant radiologists with more than 5 years’ experience working within large public hospital at that time. Visual demonstrations of real-world application of AI in practice provides an opportunity for further learning and reflection: cases to be highlighted include undetected nodules on reports (detected by AI), image quality considerations when using AI, and potential sources of AI lung nodule discrepancies.

**Table 1.** The AI software’s abbreviations and threshold values.

Pathology abbreviation	Pathology	Threshold (%)
Atl	Atelectasis	The AI software operates using a linear threshold for abnormality detection, producing an abnormality score of between 0% and 100% for common abnormalities (*see adjacent list*). The AI operating threshold was set at 15%, with a higher % signifying a higher probability of the abnormality being present. When more than 1 abnormality is listed as suspicious by the AI software, the % demonstrated on the image is for the primary abnormality, with all other abnormalities listed consecutively if they are above 15%.
Csn	Consolidation	
Fib	Fibrosis	
Ndl	Nodule	
PEf	Pleural effusion	

Abbreviation: AI = artificial intelligence.

## AI detection of lung nodules

In 2019, The National Statistics Office^10^ of England reported that LC has one of the lowest 5-year net survival estimates (<20%), which can be explained by the fact that more than 50% of cases are diagnosed at an advanced stage. This highlights the importance of early detection to improve survival rates and clinical outcomes. Undetected nodules on CXR are a recognized challenge globally, with a recent study from Thailand retrospectively identifying cancers on 78.9% of CXRs and noted that missed LC rates on CXRs in 14 previous studies ranged from 5.3% to 90.0%.^11^

**Figure 1.**
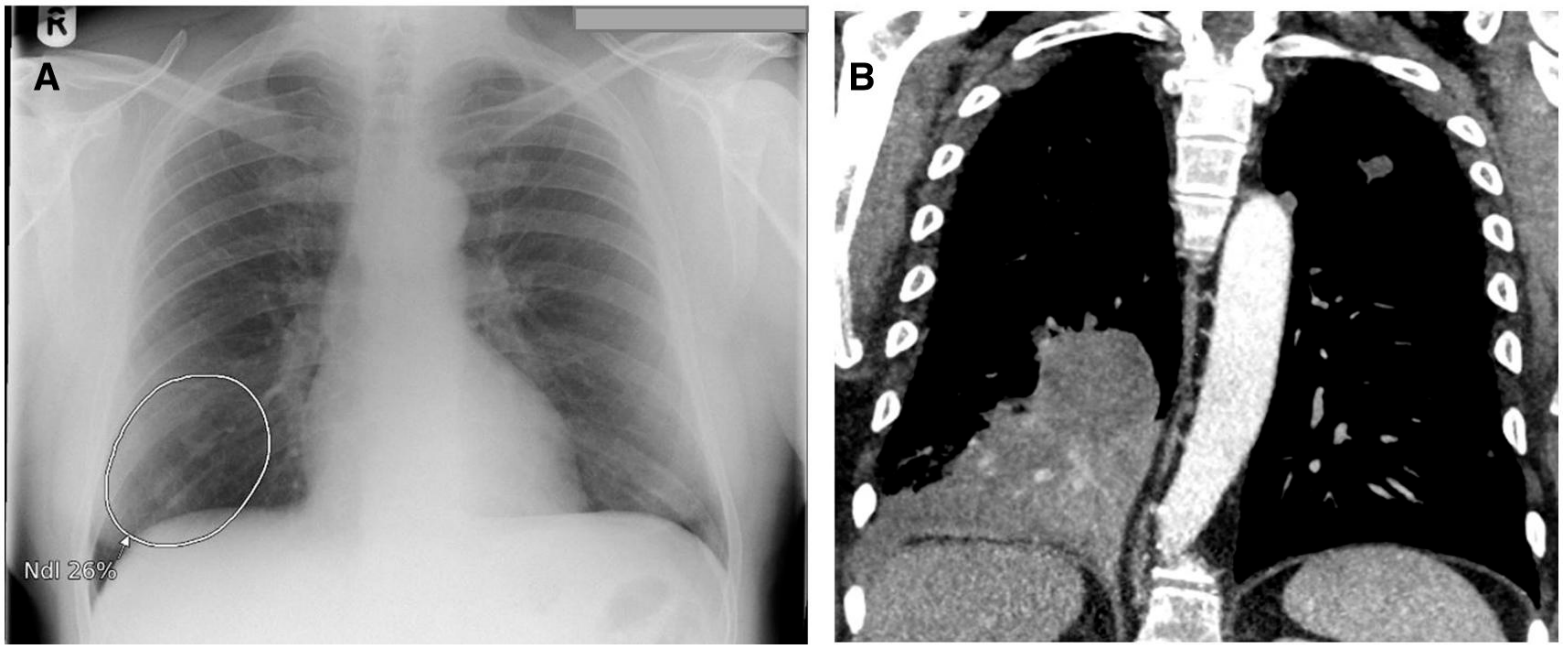
A 62-year-old male with history of smoking and shortness of breath (SOB) underwent a CXR (A), reported as no abnormility detected* (NAD). The AI software highlights suspicion of right lower lobe nodule(s) (white circle) on the same image. Three years and 10 months later, the patient has a CT chest examination (B) following episodes of haemoptysis, demonstrating a right lower lobe mass confirmed to be stage IV LC. (*Note that the NAD code for the purpose of the study denoted no solitary abnormal findings and excluded specific findings of heterogeneous diseases, that is, emphysema, interstitial lung disease). AI = artificial intelligence; CXR = chest radiograph.

**Figure 2.**
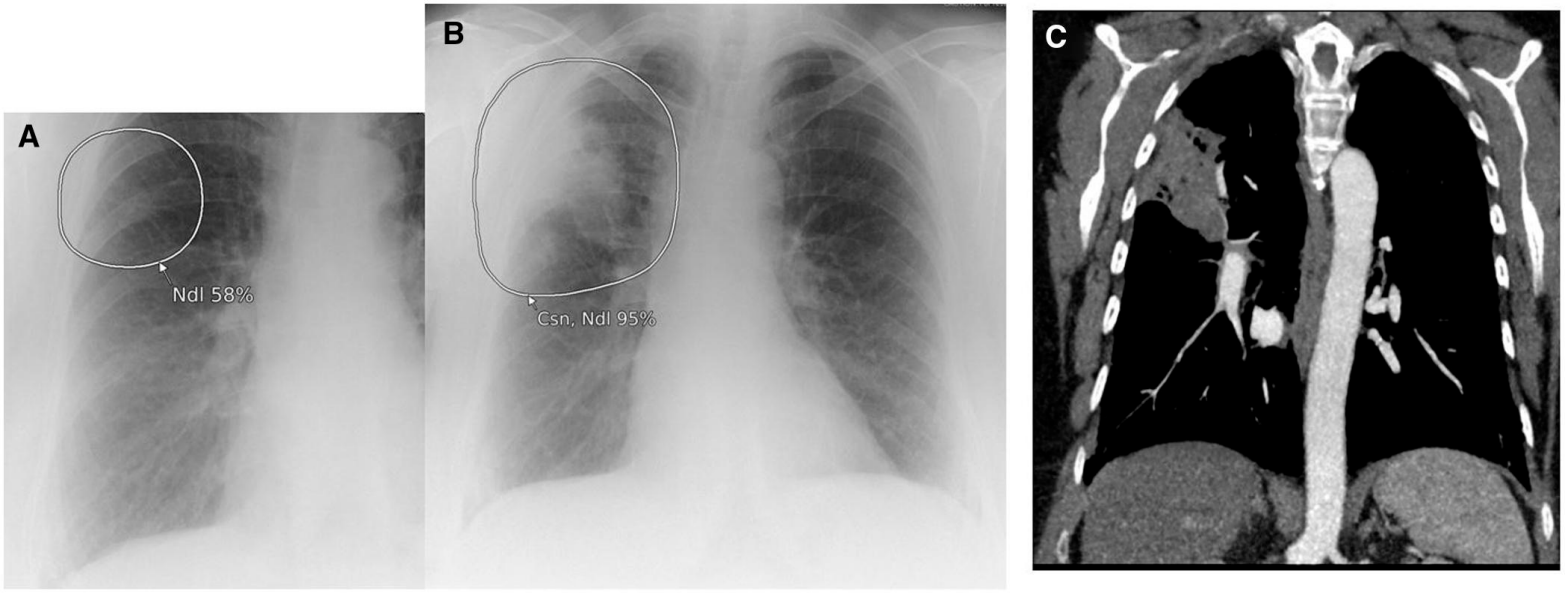
A 62-year-old female with a history of smoking and a wheezy cough underwent a CXR (A) reported as NAD. The AI software highlights suspicion of a nodule in the right upper lobe (white circle). Five years and 5 months later, the patient returns for a CXR (B) due to a persistent hoarse voice and cough, reported as likely consolidation in right upper lobe with a CT suggested to rule out mailgnancy. The AI software had similar suspicion of consolidation and/or nodule secondary on image B. A same day CT scan (C) demonstrates a right upper lobe mass, confirmed as stage IV LC. AI = artificial intelligence; CXR = chest radiograph; NAD = no abnormility detected.

**Figure 3.**
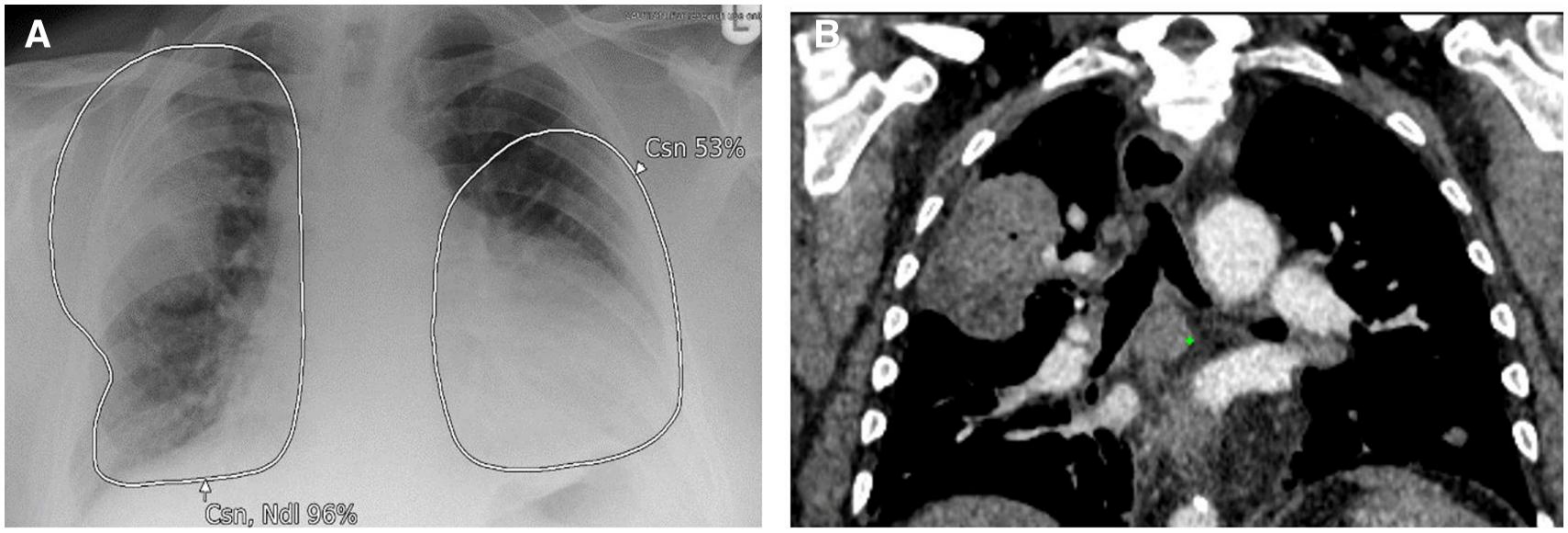
An 84-year-old male, with no history of smoking but with cardiac symptoms, underwent a CXR (A), reported as bilateral consolidation, instigating no further follow-up imaging. The AI software highlights similar suspicion of consolidation but with secondary suspicion of an underlying nodule on the right side (A, largest white circle). One year and 3 months later, the patient attends for a CT scan (B) due to progressive symptoms, which demonstrates a large right lung mass confirmed as stage IV LC. AI = artificial intelligence; CXR = chest radiograph; LC = lung cancer.

**Figure 4.**
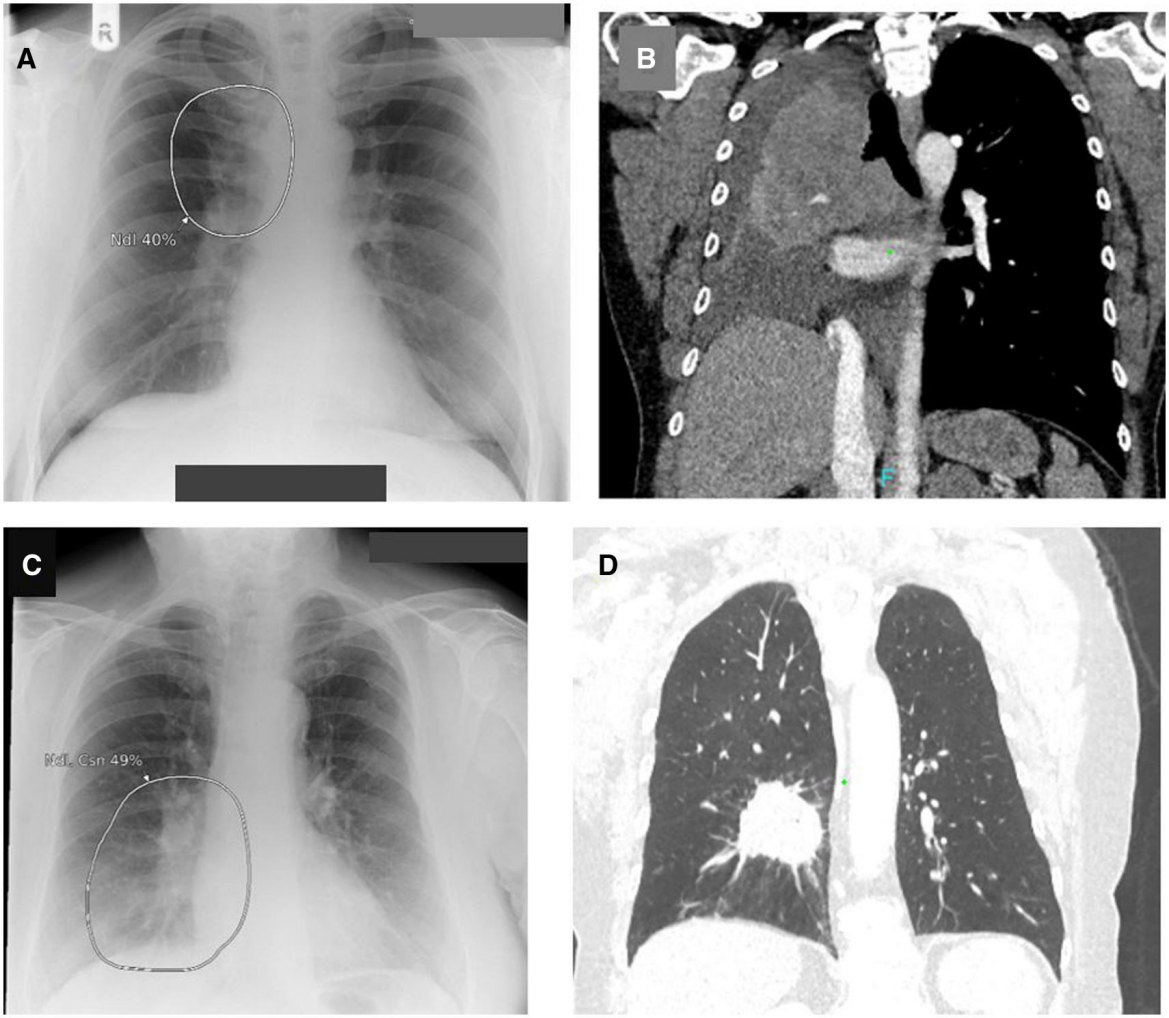
A 52-year-old male ex-smoker with a history of SOB and a cough underwent a CXR (A), reported as NAD. The AI software highlights suspicion of nodule (A, white circle) in the right perimediastinal region. One year and 1 month later, the patient attends for a CT scan (B) due to cough, SOB, and chest pain, which demonstrates a large mass confirmed as stage IV LC. An 87-year-old male smoker with history of COPD and a cough underwent a CXR (C) reported as NAD and *normal hilar shadows*. The AI software demonstrates suspicion of nodule (consolidation secondary) in the right hilar region. One year and 4 months later, the patient underwent a CT scan (D) due to an abnormal persistent cough, wheezing and phlegm that was not responding to antibiotics; this demonstrates a right hilar mass confirmed as stage IV LC. AI = artificial intelligence; CXR = chest radiograph; LC = lung cancer; NAD = no abnormility detected; SOB = shortness of breath.

**Figure 5.**
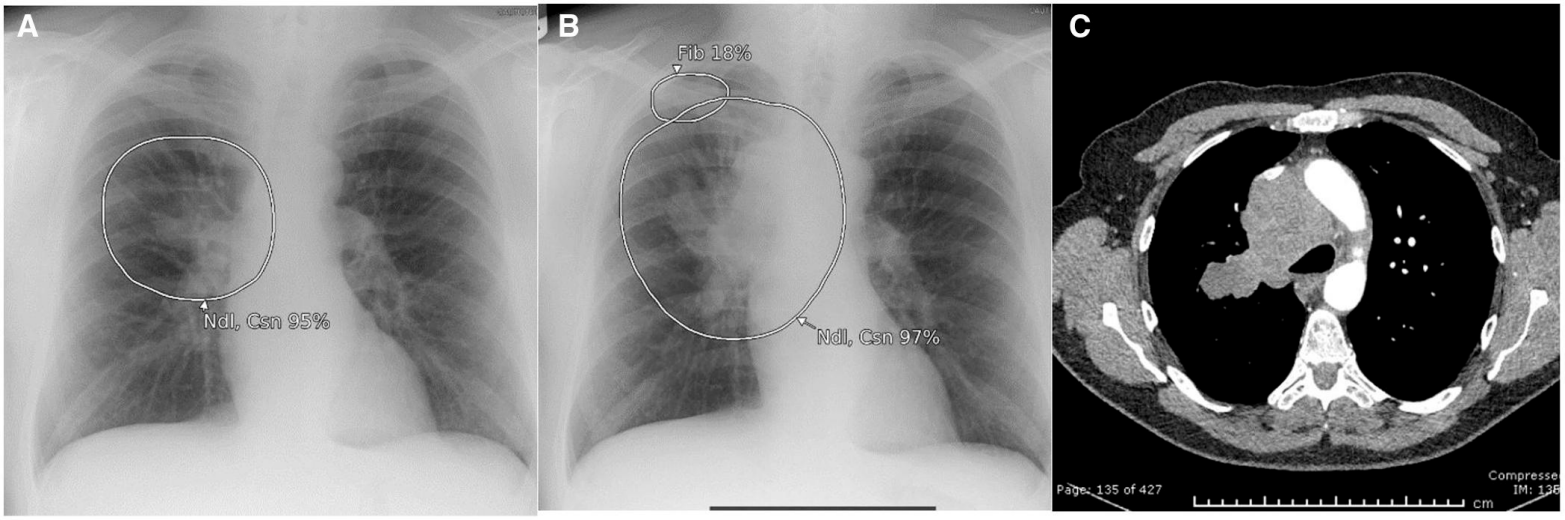
A 69-year-old male smoker with a history of haemoptysis and a cough underwent a CXR (A), report as NAD and *normal hilar shadows*. The AI software highlights a large suspicious nodule in the right hilar region. Eleven months later, the patient has another CXR (B), due to haemoptysis and weight loss. Both the report and AI software highlights suspicion of mass in the right hilar region. The patient has a subsequent CT scan (C) demonstrating a right hilar mass confirmed as stage 3b LC. AI = artificial intelligence; CXR = chest radiograph; LC = lung cancer; NAD = no abnormility detected.

**Figure 6.**
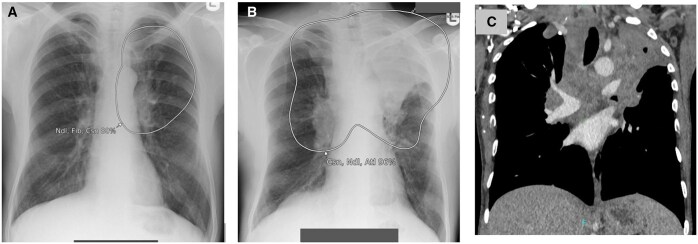
A 51-year-old male with a history of smoking, weight loss, and haemoptysis underwent a CXR (A), reported as NAD. The AI software demonstrates suspicion of a nodule in the left paramediastinal region. Ten months later, the patient returns for another CXR (B) due to persisting cough and chest pain. Both the report and AI confirm suspicion of an underlying central tumour. A subsequent CT was performed (C) demonstrating a large central left-sided mass, confirmed to be stage IV LC. AI = artificial intelligence; CXR = chest radiograph; LC = lung cancer; NAD = no abnormility detected.

**Figure 7.**
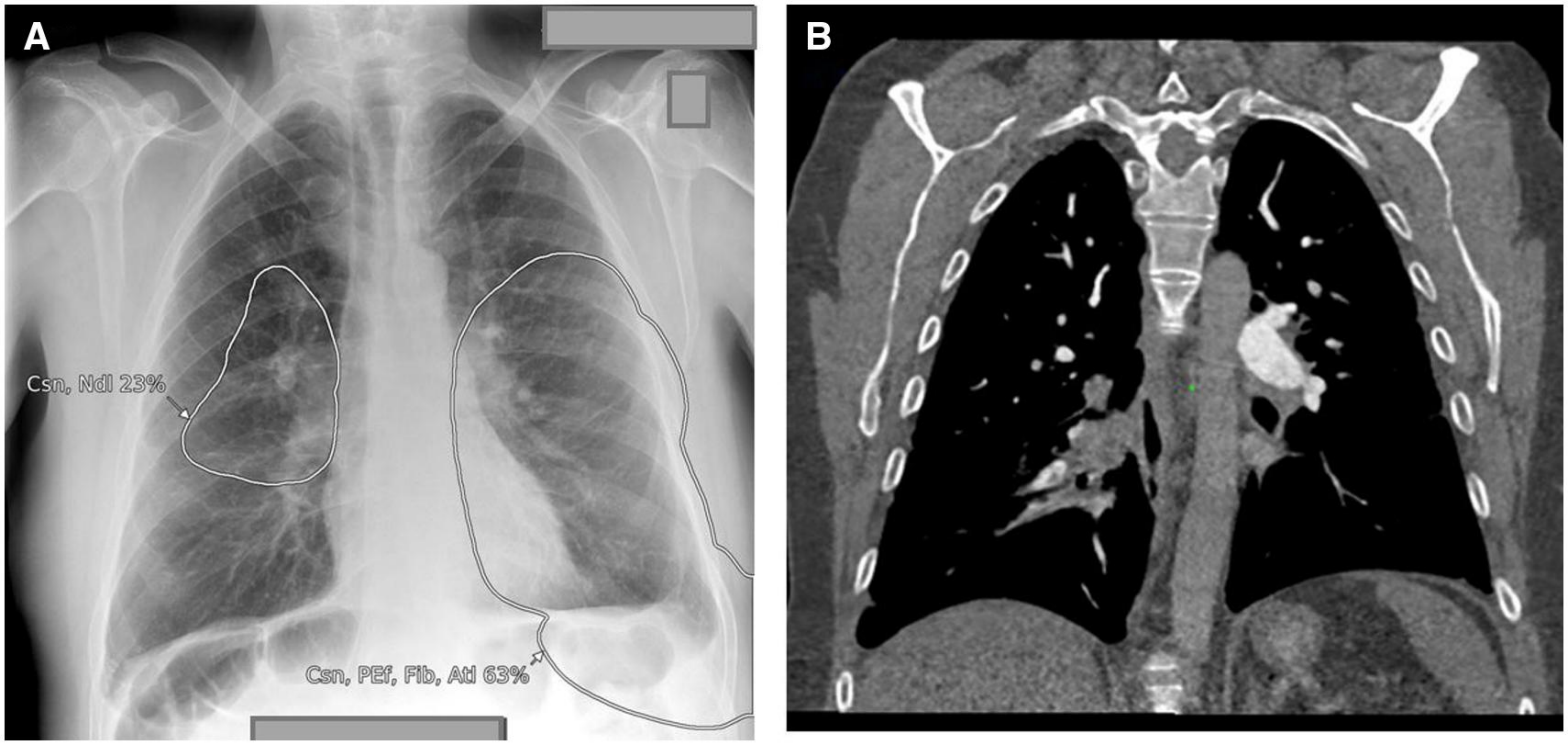
A 55-year-old male smoker with a history of cough underwent a CXR (A) reported as NAD. The AI software demonstrates suspicion of bilateral consolidation with secondary suspicion of a nodule in the right hilar region. Nine months later, a CT scan was performed (B) due to worsening SOB and productive cough, demonstrating the presence of a right hilar mass confirmed to be stage IV LC. AI = artificial intelligence; CXR = chest radiograph; LC = lung cancer; NAD = no abnormility detected; SOB = shortness of breath.

From our clinical work, there were cases where AI improved early detection of LC, with potentially significant diagnostic delay reductions as illustrated in Figures 1-3. A common area for discrepancies was within the paramediastinal and hilar regions; several nodules were detected and/or detected earlier by the AI software (Figures 4-7). This highlights that the use of AI in such cases may provide a beneficial decision-supporting tool to aid interpretations and potentially reduce the time to diagnosis of LC. Undetected paramediastinal and hilar LCs are common and align with previous works by Shimazaki et al.^4^ who found lower sensitivity when detecting LC in blind spots such as the pulmonary hila (50%-64%). These regions are complex areas to interpret radiologically due to numerous normal overlying structures, making it difficult to sometimes distinguish subtle abnormalities, especially within a bulky hilum.^12^

### AI correlation between fibrosis and nodule detection

**Figure 8.**
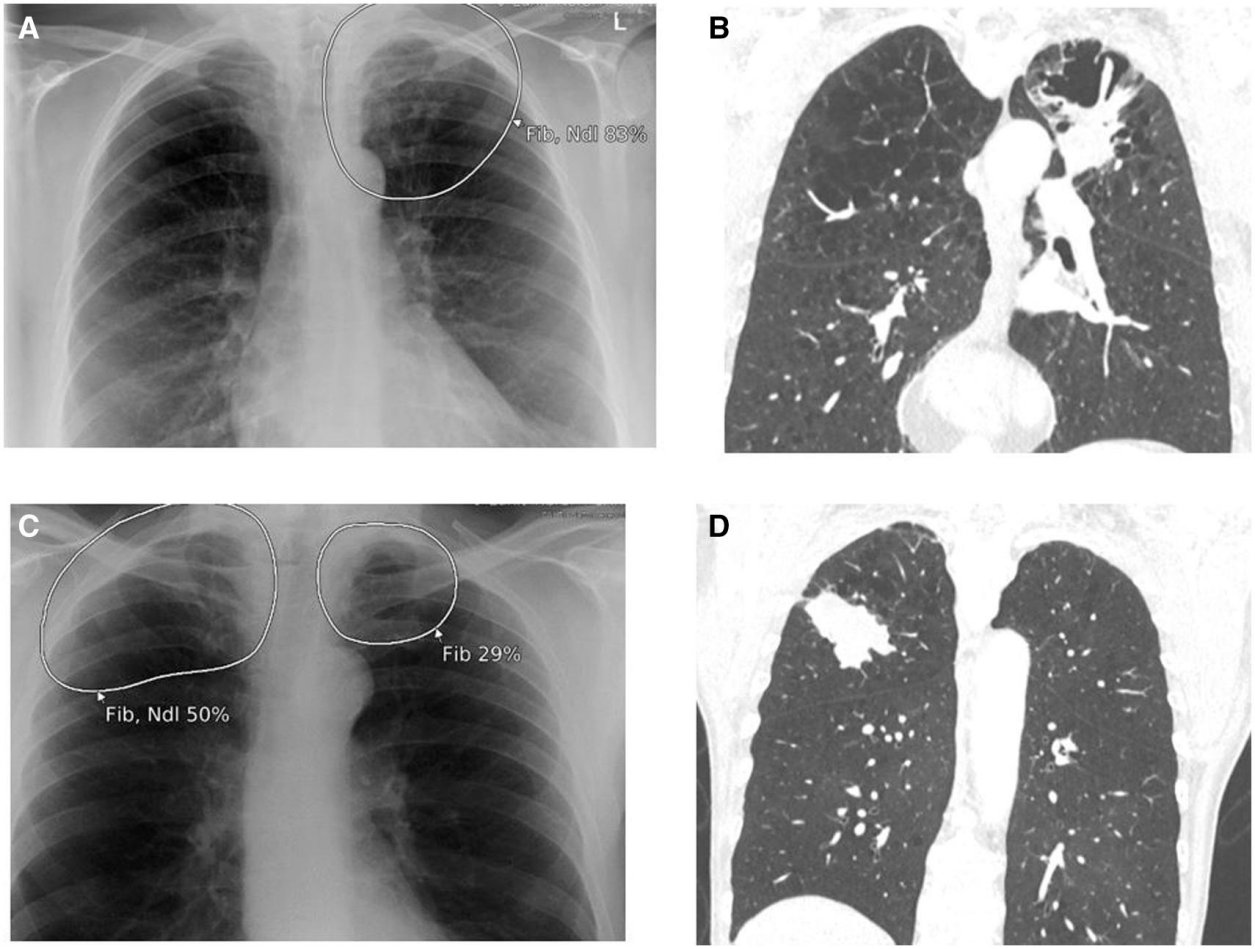
A 63-year-old female with no prior history of smoking, generally unwell, recovering from a previous chest infection, underwent a CXR (A) reported as NAD. The AI software demonstrates a suspicion of left apical fibrosis, with secondary suspicion of nodule (large white circle). Five years and 8 months later, the patient underwent a CT scan (B) as work-up for complex rheumatoid arthritis issues; this demonstrates the presence of a left apical tumour confirmed as stage IV LC. A 78-year-old male with a history of smoking and night sweats underwent a CXR (C) reported as NAD. The AI software demonstrates suspicion of bilateral apical fibrosis with secondary suspicion of a nodule on the right. Five years and 3 months later, the patient underwent a CT scan (D) due to cough and history of colon cancer, demonstrating a right upper lobe mass confirmed as stage 3 b LC. AI = artificial intelligence; CXR = chest radiograph; LC = lung cancer; NAD = no abnormility detected; SOB = shortness of breath.

**Figure 9.**
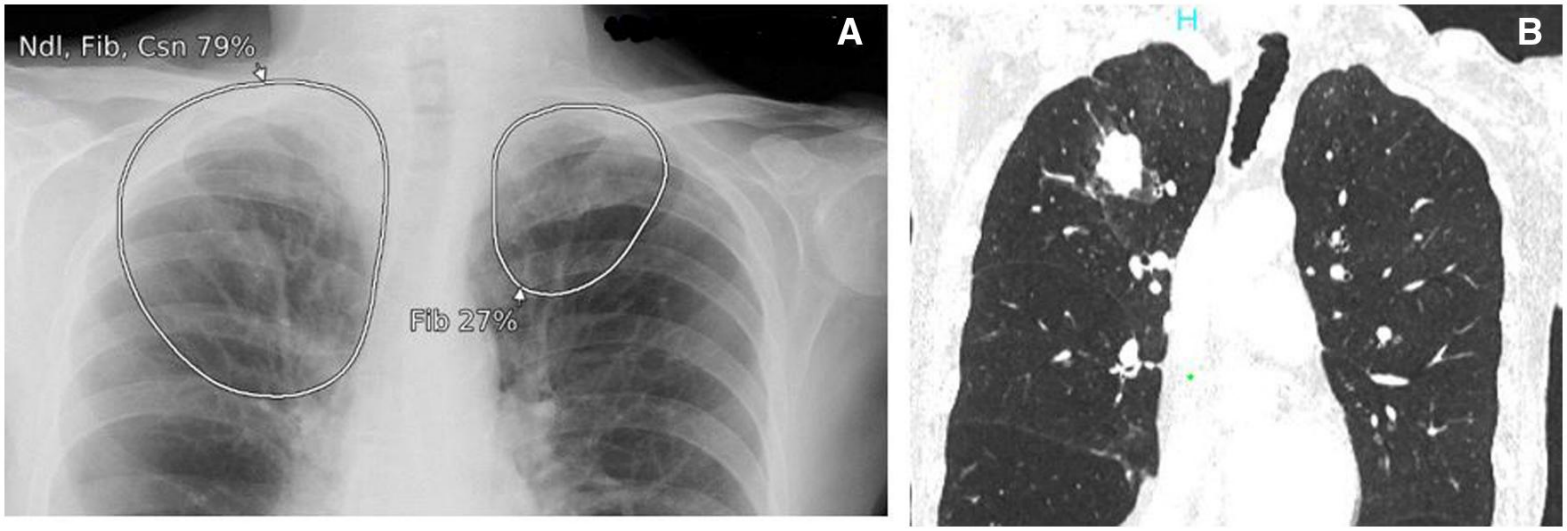
A 85-year-old male with a history of smoking and weight loss underwent a CXR (A) reported as NAD. The AI software demonstrates suspicion of a nodule in the right lung apex. One year and 2 months later, a subsequent CT scan was performed (B) due to the continuation of the same symptoms, demonstrating a right upper lobe mass, later confirmed as stage 3a LC. AI = artificial intelligence; CXR = chest radiograph; LC = lung cancer; NAD = no abnormility detected.

**Figure 10.**
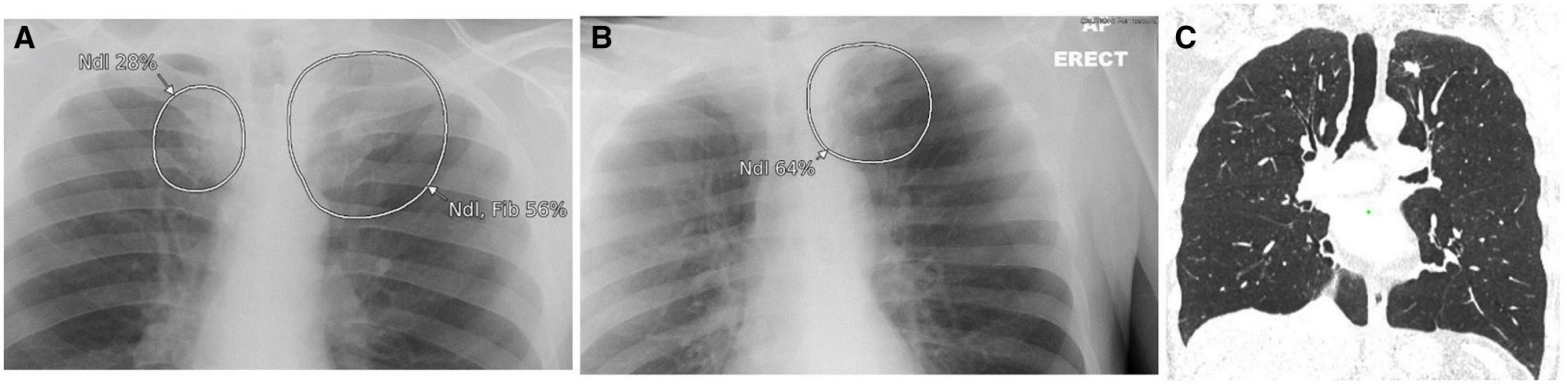
A 72-year-old male smoker with a history of COPD and a recent cough underwent a CXR (A), reported as NAD. The AI software demonstrates suspicion of bilateral nodules. Five months later, the patient returns for a subsequent CXR (B), again reported as NAD. The AI software continues to be suspicious of a nodule in the upper left lobe. Nine months later (14 month from image A), the patient underwent a CT scan (C) for weight loss and change of bowel habit, demonstrating a small left apical mass later confirmed as stage 3a LC. AI = artificial intelligence; CXR = chest radiograph; LC = lung cancer; NAD = no abnormility detected.

Although our clinical work did not aim to explore LC pathophysiology, an interesting theme identified was the potential pattern associated with the detection of fibrosis and nodules in the lung apices. There were cases (Figure 8) clinically reported as no abnormality detected; yet, the AI detected fibrosis as the primary abnormality and an apical nodule as a secondary abnormality; and a few years later, radiologically visible cancer was identified within these regions. Conversely, there were cases where the AI detected apical lung nodules and secondary suspicion of fibrosis, which later developed into LC (Figures 9 and 10). Fibrosis is often associated with an increased risk of developing LC, but also the appearances of fibrosis can mimic cancer (and vice versa). This is why there might be a secondary output score for both pathologies, respectively.^12^ In addition, lung apices, similar to the hilar and paramediastinal regions, is a common radiological blind spot, with missed LCs reported within this region.^12^

## AI FP cases

False positive findings are a well-known phenomenon in radiology, which may lead to unnecessary follow-up investigations. FPs also occur when using AI, which is one of the main concerns associated with clinical deployment.^7^ Bernstein et al.^7^ found that providing radiologists with appropriate training, whilst ensuring they anticipate FPs and false negatives, would reduce the extent to which they are influenced by incorrect results. A better understanding of AI discrepancies would help radiologists use AI more effectively and avoid the potential for over-reliance on such technology. Strubchevska et al.^13^ suggests that a lack of transparency in AI decision-making processes makes it challenging for radiologists to understand the rationale behind certain outputs. This is further reinforced by the new RCR AI Guidance (2024);^6^ this recommends that future studies should focus on the importance of observer decision-making and how AI affects this, especially surrounding difficult cases of discrepancies.

**Figure 11.**
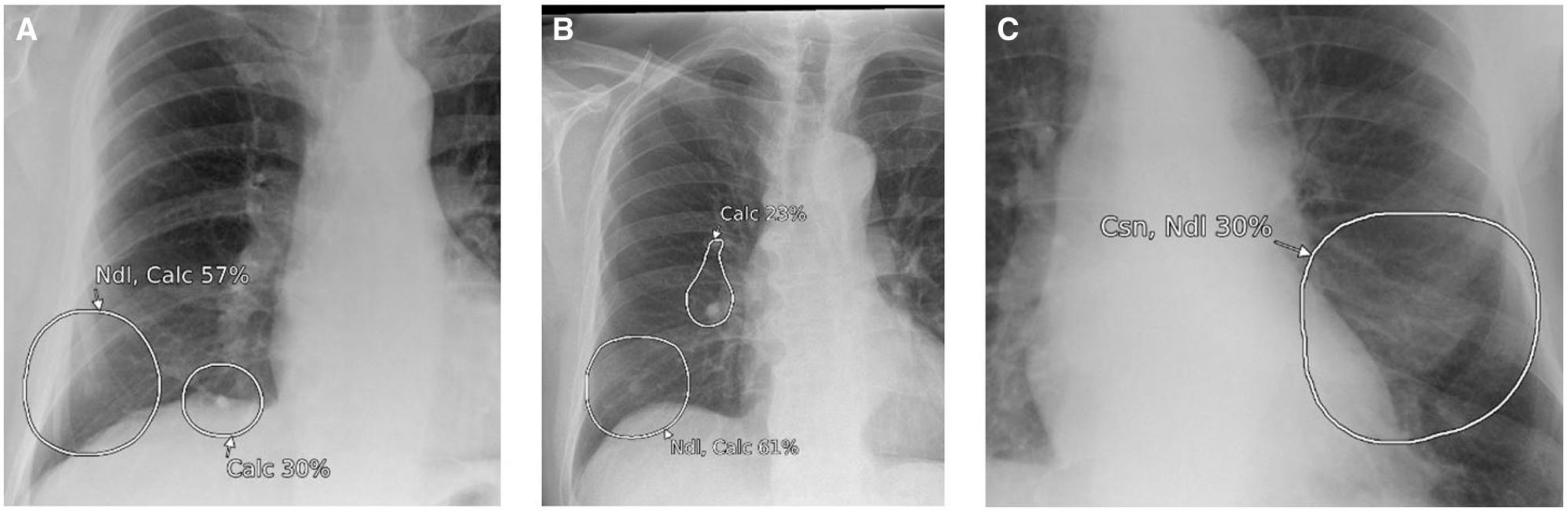
Images (A) and (B) demonstrate the AI software’s suspicion of a nodule (large circles in both images). Following independent review by a consultant radiologist, these were confirmed to be nipple shadows (*the smaller circles suspicious of calcification were considered true positives*). Image (C) also demonstrates the AI software suspicious of consolidation and a secondary nodule adjacent to the heart, which, following independent review by a consultant radiologist, was confirmed to be breast shadow. AI = artificial intelligence.

**Figure 12.**
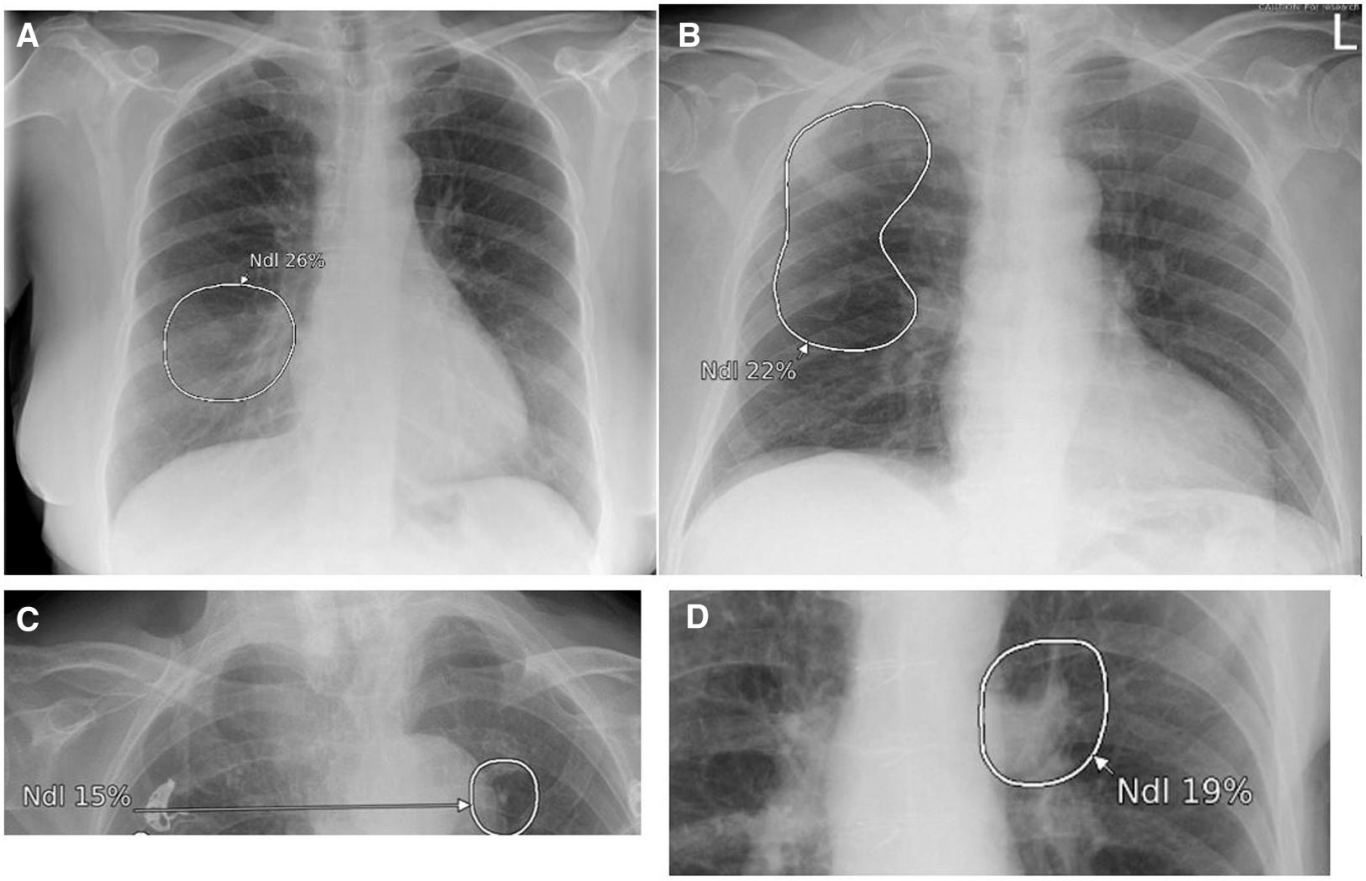
Image (A) and (B) demonstrates the AI software’s suspicion of nodules, but on independent review by a consultant radiologist, they were confirmed to be a companion shadow (A) and scapula shadow (B). Images (C) and (D) also demonstrates the AI software’s suspicion of nodules, but on independent review by a consultant radiologist, they were confirmed to be end-on vessels. AI = artificial intelligence.

A re-review of our AI discrepancies by an independent consultant radiologist revealed common causes of FPs. These included “shadows” from various structures, such as the nipple and breast (Figure 11), scapula and companion (Figure 12A and B), and end-on blood vessels (Figure 12C and D). No restrictions were placed on radiologists when re-reviewing these discrepancies. Futures studies should consider the role of “the holistic approach” to image interpretation when classifying these as FP. Radiologists typically would consider a patient’s history, symptoms, laboratory results, and prior imaging studies when interpreting an image; essential elements for correct diagnosis.^14^ This emphasizes the importance of human oversight and demonstrates the role of AI as a decision-support tool.

## Influence of image quality on AI performance

**Figure 13.**
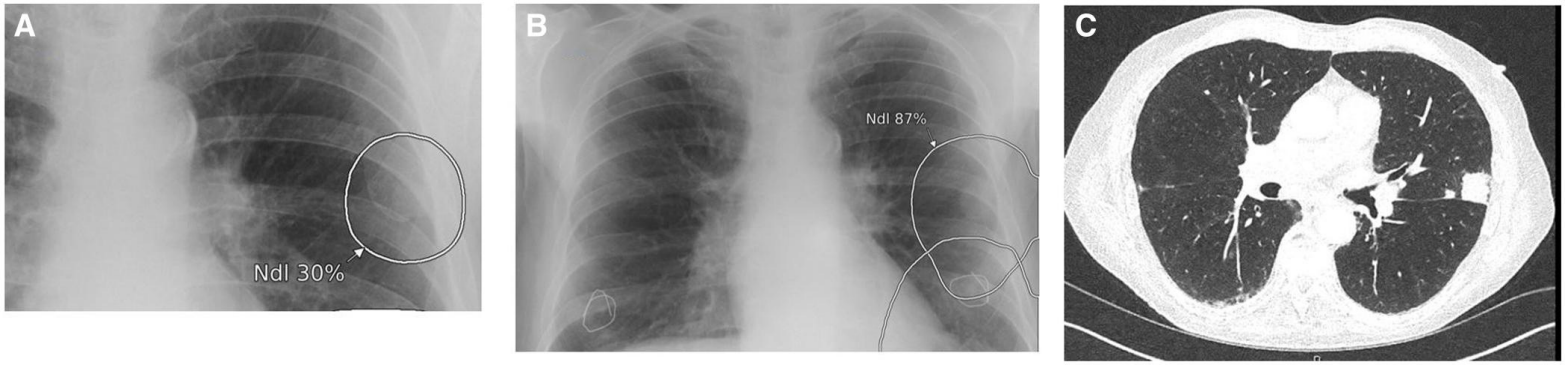
An 85-year-old male with a history of productive cough and rigors underwent a CXR (A), reported as NAD. The AI software demonstrates suspicion of a nodule on the left side of the chest periphery, behind the scapula, and ribs. Six months later, the patient returns for another CXR (B) due to being unwell, whereby the report and AI software are suspicious of a nodule on the left side. Note that the scapula is removed from the lung field, making it easier to visualize the nodule, also note nipple markers on the image (B). A CT scan is subsequently performed (C) demonstrating a left-sided mass confirmed as stage 3b LC. AI = artificial intelligence; CXR = chest radiograph; LC = lung cancer; NAD = no abnormility detected.

Recent AI studies for LC detection on CXR often use strict inclusion and exclusion criteria for selecting images, for example, the use of posterior–anterior CXRs only,^1^^,^^5^ and excluding nodules of certain measurements,^3^^,^^5^ or images that contained other pathologies mimicking tumours.^3^^,^^5^ This does not provide “real-world” evaluations of AI performance, making it difficult to translate and validate findings for clinical implementation. It is important to understand how AI performance is affected by image quality or the presence of medical devices. York et al.^14^ suggests devices often introduce artefacts and obscure underlying anatomy, which complicates image interpretation. Our cases demonstrated that common metallic artefacts did not hinder or cause AI misinterpretation of ECG tabs and leads (Figures 12C and D), midline sternotomy wires, and nipple markers (Figure 13B).

**Figure 14.**
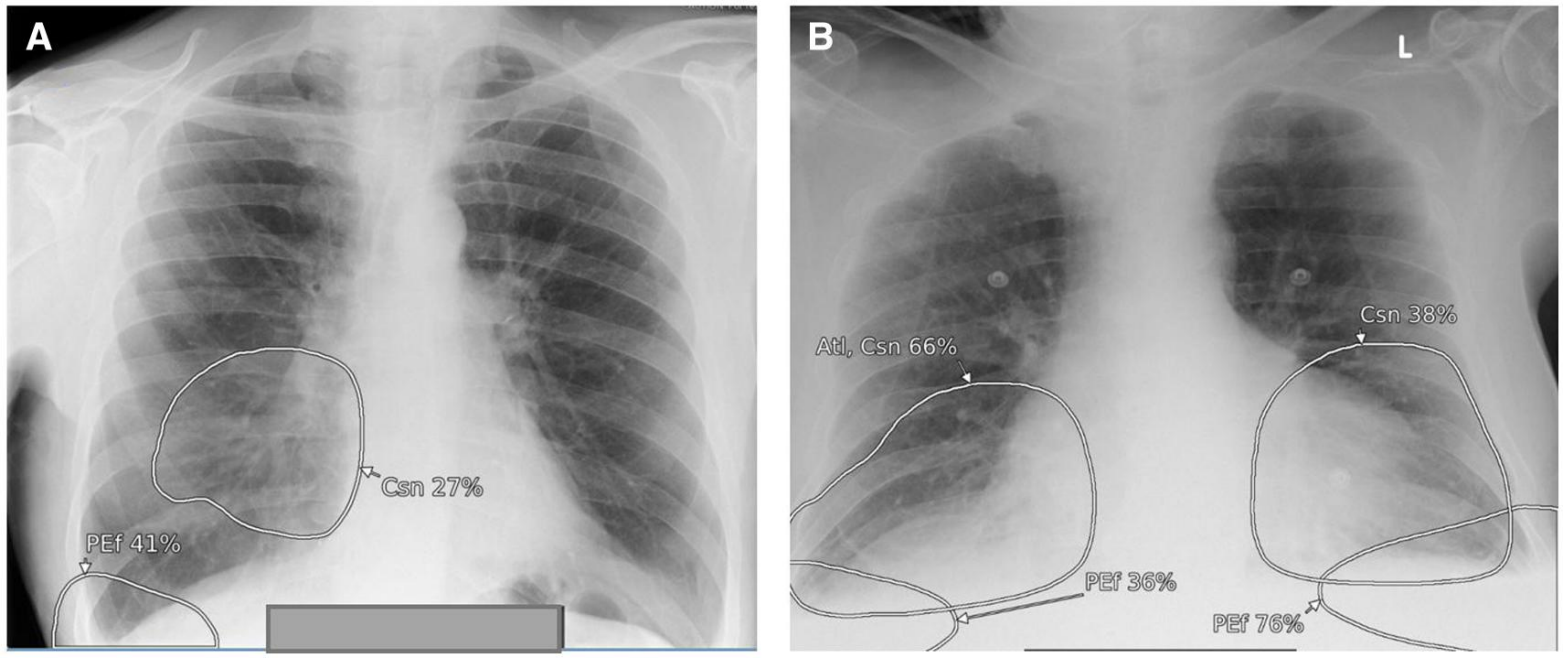
Images (A) and (B) demonstrate the AI software’s suspicion of a pleural effusion (PEf), consolidation (Csn), and atelectasis (Atl) in a control “normal” patient. On independent review by a consultant radiologist, it was confirmed to be an underexposed image. Note on image (B), the presence of some cardiac monitoring tabs. AI = artificial intelligence.

**Figure 15.**
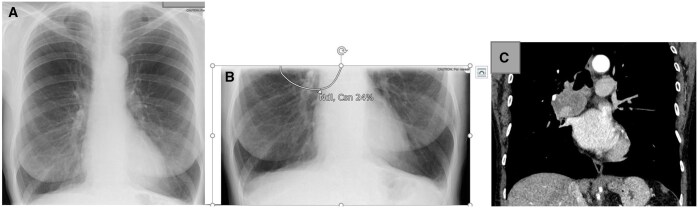
A 71-year-old female ex-smoker with increasing breathlessness underwent a CXR (A). Due to the lung bases being omitted from this image, a second lung bases X-ray image was acquired (B). Both images (A and B) were reported as NAD, with the AI software being NAD for image (A) but suspicious of a nodule on the subsequent image (B) only. Ten months later, the patient returns for a CT scan (C) due to haemoptysis, demonstrated a right hilar mass later confirmed to be stage IV LC. AI = artificial intelligence; CXR = chest radiograph; LC = lung cancer; NAD = no abnormility detected.

Another interesting insight is illustrated in Figure 12B; this demonstrates a scapulae shadow being mistaken for a nodule/consolidation. When undertaking a CXR, one of the radiographic quality criteria is to clear the scapulae from the lung fields. When this is not achieved, it may lead to interpretation issues (Figure 13). Robert et al.^15^ reported multiple missed LC on prior CXRs with lesions often obscured by bony anatomy. The importance of exposure factors for optimal penetration and contrast is another image quality consideration. Figure 14 demonstrates where the AI suspects a pleural effusion, but on re-review, it was re-classified as an underpenetrated CXR, mimicking the appearances of basal pathology. Exposure factors, coverage, and zooming could also explain why the AI has identified a lung nodule on a smaller field of view of the lung bases and not on the initial CXR (Figure 15). The above reaffirms and highlights the importance of high-quality imaging for optimal AI performance and reinforces that future studies should include a sample of images representative of clinical practice.

## Conclusion

Given the frequency that CXRs are performed and their susceptibility to interpretation discrepancies, the use of AI as a decision support tool may complement human expertise, improving the overall performance of CXR interpretation in real-world clinical practice. However, it is important to have awareness of potential discrepancies relating to the use of AI. Cases within this review provide a visual portrayal of how a commercially available AI tool provided opportunities to detect and reduce interpretation errors. It also demonstrates potential common sources of AI FPs and the possible influence of image quality on AI performance. Enhancing the knowledge and learning of AI users about the capabilities and weaknesses associated with AI is essential, whilst highlighting the importance of local clinical validation prior to adoption. This review can also help inform and direct future research by highlighting the importance of AI software clinical training and an initial AI sandbox to help manage and learn from AI discrepancies, which may maximize its benefits while minimizing the risk of automation bias. Future studies should also focus on the importance of observer decision-making and how AI may affect this, especially surrounding difficult cases of discrepancies.

## Notes

**NOTE.—**The AI software was used retrospectively on historic CXR images, which were already clinically reported, and therefore results from the AI were not available when the CXRs were initially acquired and formally reported.
